# Cadaveric White Matter Dissection Study of the Telencephalic Flexure: Surgical Implications

**DOI:** 10.3389/fneur.2022.757757

**Published:** 2022-02-15

**Authors:** Pablo González-López, Giulia Cossu, Cynthia M. Thomas, Jeffery S. Marston, Cristina Gómez, Etienne Pralong, Mahmoud Messerer, Roy T. Daniel

**Affiliations:** ^1^Hospital General Universitario de Alicante, Alicante, Spain; ^2^University Hospital of Lausanne, University of Lausanne, Lausanne, Switzerland; ^3^Charles University, Prague, Czechia

**Keywords:** brain surgery, disconnective surgery, epilepsy surgery, functional connectome, oncology, telencephalic flexure, sylvian fissure

## Abstract

Neurosurgery has traditionally been overtly focused on the study of anatomy and functions of cortical areas with microsurgical techniques aimed at preserving eloquent cortices. In the last two decades, there has been ever-increasing data emerging from advances in neuroimaging (principally diffusion tensor imaging) and clinical studies (principally from awake surgeries) that point to the important contribution of white matter tracts (WMT) that influence neurological function as part of a brain network. Major scientific consortiums worldwide, currently working on this human brain connectome, are providing evidence that is dramatically altering the manner in which we view neurosurgical procedures. The development of the telencephalic flexure, a major landmark during the human embryogenesis of the central nervous system (CNS), severely affects the cortical/subcortical anatomy in and around the sylvian fissure and thus the different interacting brain networks. Indeed, the telencephalic flexure modifies the anatomy of the human brain with the more posterior areas becoming ventral and lateral and associative fibers connecting the anterior areas with the previous posterior ones follow the flexure, thus becoming semicircular. In these areas, the projection, association, and commissural fibers intermingle with some WMT remaining curved and others longitudinal. Essentially the ultimate shape and location of these tracts are determined by the development of the telencephalic flexure. Five adult human brains were dissected (medial to lateral and lateral to medial) with a view to describing this intricate anatomy. To better understand the 3D orientation of the WMT of the region we have correlated the cadaveric data with the anatomy presented in the literature of the flexure during human neuro-embryogenesis in addition to cross-species comparisons of the flexure. The precise definition of the connectome of the telencephalic flexure is primordial during glioma surgery and for disconnective epilepsy surgery in this region.

## Introduction

**Figure 1:**
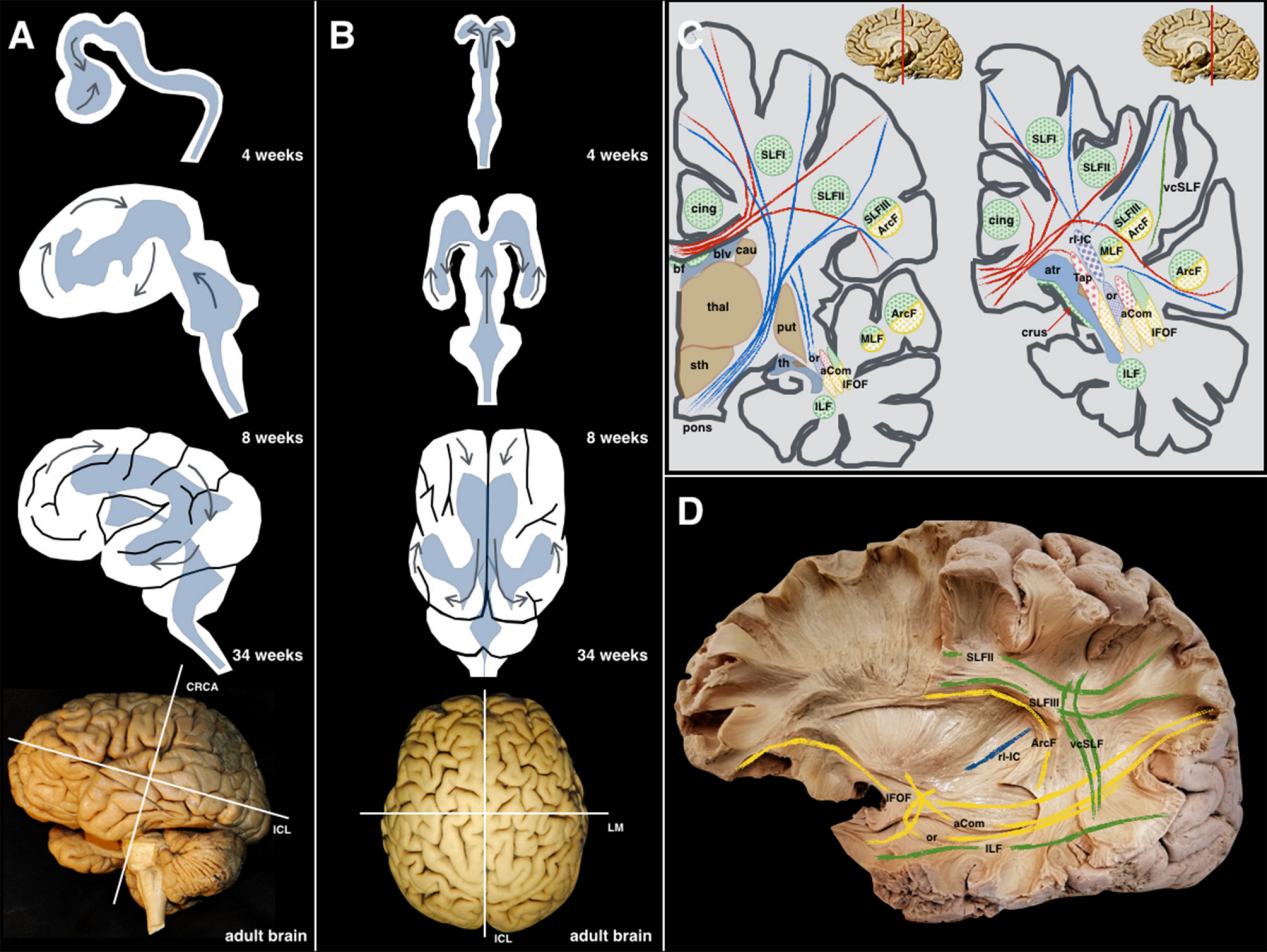
**(A)** Lateral perspective: The different steps during the embryological development (4, 8, 34 weeks, and adult brain) of the human brain are shown along with the forces that produce morphological stress *(arrows)*. The main internal forces push the occipital lobe from a dorsal to a posterior location resulting in the temporal lobe moving from its posterior and medial location to a more anterior and lateral one. The telencephalic flexure is the result of all these movements happening during the early embryological formation of the CNS. **(B)** Superior perspective: the two telencephalic vesicles are shown as well as the early ventricular cavities. For an accurate understanding of the superficial and deep anatomy, the three planes of the space have been defined according to Talairach's description: the cerebral space is defined by the anteroposterior or intercommissural line *(ICL)* between the anterior and posterior commissures, the craniocaudal *(CRCA)* between vertex and midbrain and perpendicular to the ICL line, and the lateromedial *(LM)* between both hemispheres, and perpendicular to the previous lines. **(C)** As a result of the complex shape acquired by the human brain, and the 3D organization of the ventricular system, most of the association fibers in this area show a curved shape with a specific arrangement pattern. The purely latero-medially oriented fibers are represented in *red*; the craneo-caudal fibers in *blue*; the anteroposterior fibers are *green*; the *yellow* color is employed to represent those fibers that being latero-medial, anteroposterior, or craneo-caudal, that change its direction at some point, becoming curved in shape. **(D)** These anatomical relationships are shown and partially schematized in this left hemisphere showing longitudinally oriented association fibers *(green)*, curved-shape association fibers *(yellow)*, and purely longitudinal projection fibers *(blue)*. aCom, anterior commissure; atr, atrium; ArcF, arcuate fascicle; bf, body of fornix; blv, body of the lateral ventricle; cau, caudate nucleus; cing, cingulum; crus, crus of the fornix; IFOF, inferior frontooccipital fascicle; ILF, inferior longitudinal fascicle; MLF, middle longitudinal fascicle; or, optic radiations; pons; brainstem protuberance; put, putamen; rl-IC, retrolenticular component of the Internal Capsule; SLF I, Superior Longitudinal Fascicle I; SLF II, Superior Longitudinal Fascicle II; SLF III, Superior Longitudinal Fascicle III; sth, subthalamic area; Tap, tapetum; th, temporal horn; thal, thalamus; vcSLF, vertical component of the SLF.

Neurosurgical interventions in and around the perisylvian cortices often represent a surgical challenge due to the complexity of the sulcogyral architecture and underlying fiber systems. The capricious anatomy of this region is principally related to the development of the telencephalic flexure, which represents a major landmark during human neuro-embryogenesis (Figure 1).

During the early embryonic stages, the telencephalic hemispheres bend caudally in a ventral and rostral direction, resembling a “C-shaped” structure. Therefore, the upcoming temporal lobe derives from the posterior pole of the primitive telencephalon, while the occipital lobe is derived from its dorsal wall. This folding of the telencephalic hemispheres (telencephalic flexure) becomes the operculum and the future Sylvian fissure. Following a complex interaction and excitatory/inhibitory neural signaling, this folding will finally delineate the curved shape of most of the fibers connecting the implicated cortical regions. As a consequence of the folding of the telencephalic hemispheres, the fibers' final shape “suffer” the effects of the aforementioned flexion and the functional neuroanatomy of the connectome in this area may be challenging to understand.

The aim of this study is to illustrate the white matter anatomy around the telencephalic flexure along with a comprehensive review of the mechanisms involved in its embryological development and with the help of a cross-species analysis, and how the understanding of this complex anatomical evolvement might help surgeons to optimize surgical interventions around this region. The subcortical connectivity should be maximally preserved through the performance of selective procedures to preserve the neurological and cognitive functions of our patients.

## Methods

In this study, five cadaveric human brains were prepared following Klingler's technique. The specimens were extracted during the first 12 h postmortem and placed in 10% formalin solution for 8 weeks. The brains were washed under running water and frozen to −15°C for 2 weeks (1). The freezing allowed the expansion of the extracellular water allowing the spreading of the fibers (2). After de-freezing the brains, dissection was carried out in a lateral-to-medial and medial-to-lateral direction. A transventricular dissection was finally carried out, with the aim of a deeper understanding of the aforementioned fibers. The main white matter tracts were identified according to the surface anatomy, and their direction was described in the three planes of the space (3) (Figure 1).

In order to better sustain the results based on the brain dissections, as well as the literature review, two surgical cases were discussed to illustrate the importance of this anatomic understanding. Both patients gave their consent to share their clinical records.

## Results

### Lateral-to-Medial Dissection

#### Frontal Region

**Figure 2:**
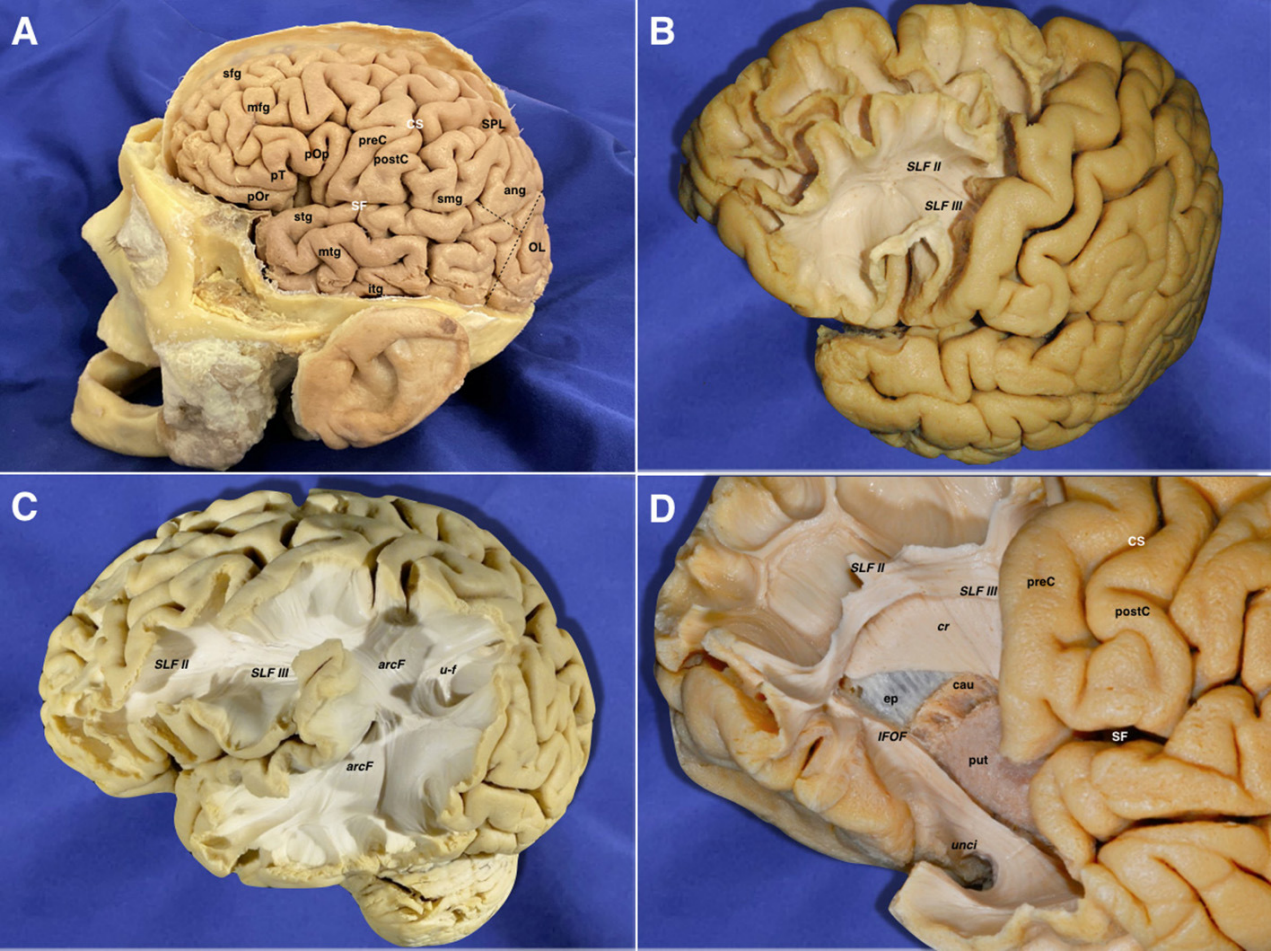
**(A)** Lateral view of a left hemisphere (before the brain's extraction from the skull). The central lobe represents the boundary between the frontal and parietal areas. The pre-occipital notch and distal end of the sylvian fissure helped to delineate the indistinct border between the temporal, parietal, and occipital lobes. This is the most important pivotal point resulting from the different forces that affect the growth of the human brain during its embryological development. **(B)** The frontal lobe in the left hemisphere has been partially dissected anterior to the precentral sulcus. The gray matter has been scraped away showing the u-fibers in the superior frontal gyrus. The inferior frontal gyrus has been removed along with the underlying u-fibers in this area, showing a clear view of the SLF II & SLF III frontal projections. **(C)** The cortex and u-fibers have been removed showing the SLF II & SLF III and their close relationship with the arcuate fascicle wrapping around the posterior Sylvian point. U-fibers are shown connecting the superior parietal lobe and the angular gyrus. **(D)** A more advanced step in the frontal dissection is shown. The projections of the anterior limb of the internal capsule are arising in between the caudate nucleus and putamen, just lateral to the ependyma of the lateral ventricle frontal horn. The internal capsule cranial projections intermingle with the lateral projections of the corpus callosum at this point, forming a longitudinal layer of tracts, which runs perpendicular in direction to the long association ones (SLF II & III). The temporal pole fibers are also exposed after removing their u-fibers. The inferior fronto-occipital fascicle and uncinate fascicle are also exposed in the temporal aspect. Ang, angular gyrus; arcF, arcuate fascicle; cau, caudate nucleus; cr, corona radiata; CS, central sulcus; ep, ependyma; IFOF, inferior frontooccipital fascicle; itg, inferior temporal gyrus; mfg, middle frontal gyrus; mtg, middle temporal gyrus; OL, occipital lobe; pOp, pars opercularis; pOr, pars orbitalis; postC, postcentral gyrus; preC, precentral gyrus; pT, pars triangularis; put, putamen; SF, sylvian fissure; sfg, superior frontal gyrus; SLF II, superior longitudinal fasciculus II; SLF III, superior longitudinal fasciculus III; smg, supramarginal gyrus; SPL, superior parietal lobe; stg, superior temporal gyrus; u-f, u-fibers; unci, uncinate fasciculus.

The removal of the cortex and the u-fibers exposed the superior longitudinal fasciculus II (SLF II) connecting parietal and frontal lobes, and the SLF III between the inferior frontal and supramarginal gyri were found running in an anteroposterior linear direction. After removing the insular cortex, extreme and external capsules, the lateral aspect of the putamen was uncovered. Deeper to the lentiform nucleus, the internal capsule (IC) was seen medially and projecting upwards intermingling with the corpus callosum (CC) lateral projections forming together the corona radiata. The anterior limb and genu of the IC were longitudinally oriented into the vertical plane (Figure 2).

#### Parieto-Occipito-Temporal Region

**Figure 3:**
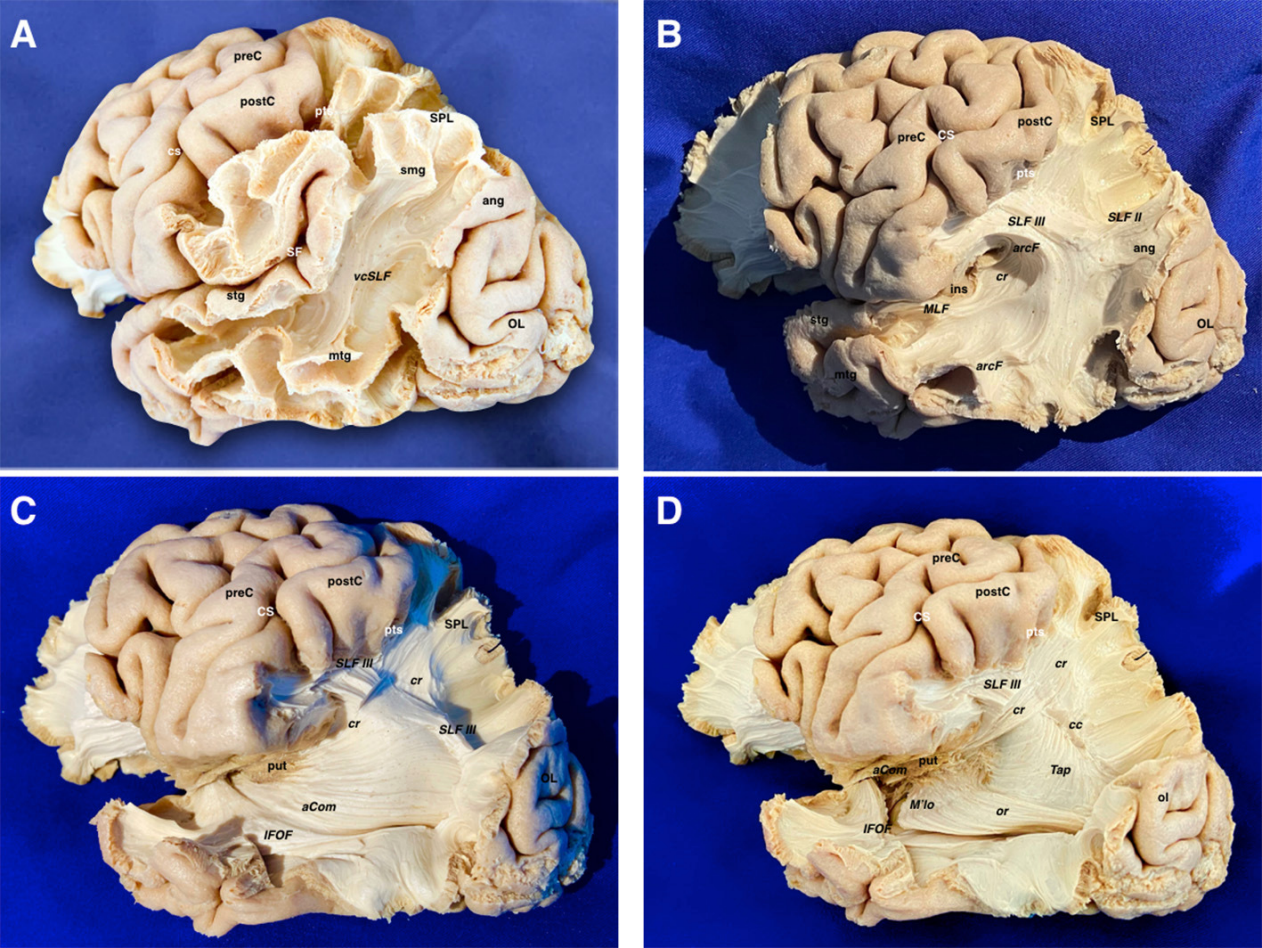
**(A)** The temporo-parieto-occipital dissection starts by scraping away the cortex exposing the u-fibers and the most superficial long association fiber tract in this region, which is the vertical component of the SLF connecting the superior parietal lobe and supramarginal gyrus with the posterior aspect of the middle and superior temporal gyri. It is interesting to note that this is the only vertically oriented association bundle. **(B)** The vertical component of SLF has been peeled off to expose the next layer of tracts in this area. Thus, the arcuate fascicle is seen wrapping around the posterior insular point. The most posterior part of the SLF II and SLF III are shown just dorsal to the curve of the arcuate fascicle. The middle longitudinal fascicle is shown arising from the middle third of the superior temporal gyrus. Running posteriorly, the MLF is situated just underlying the arcuate. In a deeper location, the retro-lenticular aspect of the internal capsule is shown being part of the corona radiata. **(C)** The AF, SLF II & III, have been cut dorsally at the level of the supramarginal gyrus, uncovering the posterior extension of the IFOF, retro-lenticular fibers of the IC projecting to the parietal and occipital lobes, and the posterior portion of the lateral extension of the anterior commissure (AC) connecting both temporo- occipital basal lobes. The MLF has been removed showing the retro- and sublenticular projections of the IC in relation to the putamen. In the temporal side, the most external layers of the sagittal stratum (anterior commissure and inferior fronto-occipital fascicle) are dissected arising from the ventral aspect of the telencephalic flexure area. **(D)** Scraping away the corona radiata into the posterior part of the flexure has exposed the tapetum just lateral to the ependyma of the lateral ventricle atrium. In the ventral aspect of the telencephalic flexure, the anterior commissure has been cut and the posterior extension of the IFOF is removed. This maneuver allows uncovering the optic radiations, which is the deeper layer of the sagittal stratum. aCom, anterior commissure; ang, angular gyrus; arcF, arcuate fascicle; cr, corona radiata; CS, central sulcus; IFOF, inferior frontooccipital; ins, insula; MLF, middle longitudinal fasciculus; mtg, middle temporal gyrus; OL, occipital lobe; postC, postcentral gyrus; preC, precentral gyrus; pts, postcentral sulcus; put, putamen; SF, Sylvian fissure; SLF II, superior longitudinal fasciculus II; SLF III, superior longitudinal fasciculus III; smg, supra marginal gyrus; SPL, superior parietal lobe; stg, superior temporal gyrus; vcSLF, vertical component of the superior longitudinal fasciculus.

**Figure 4:**
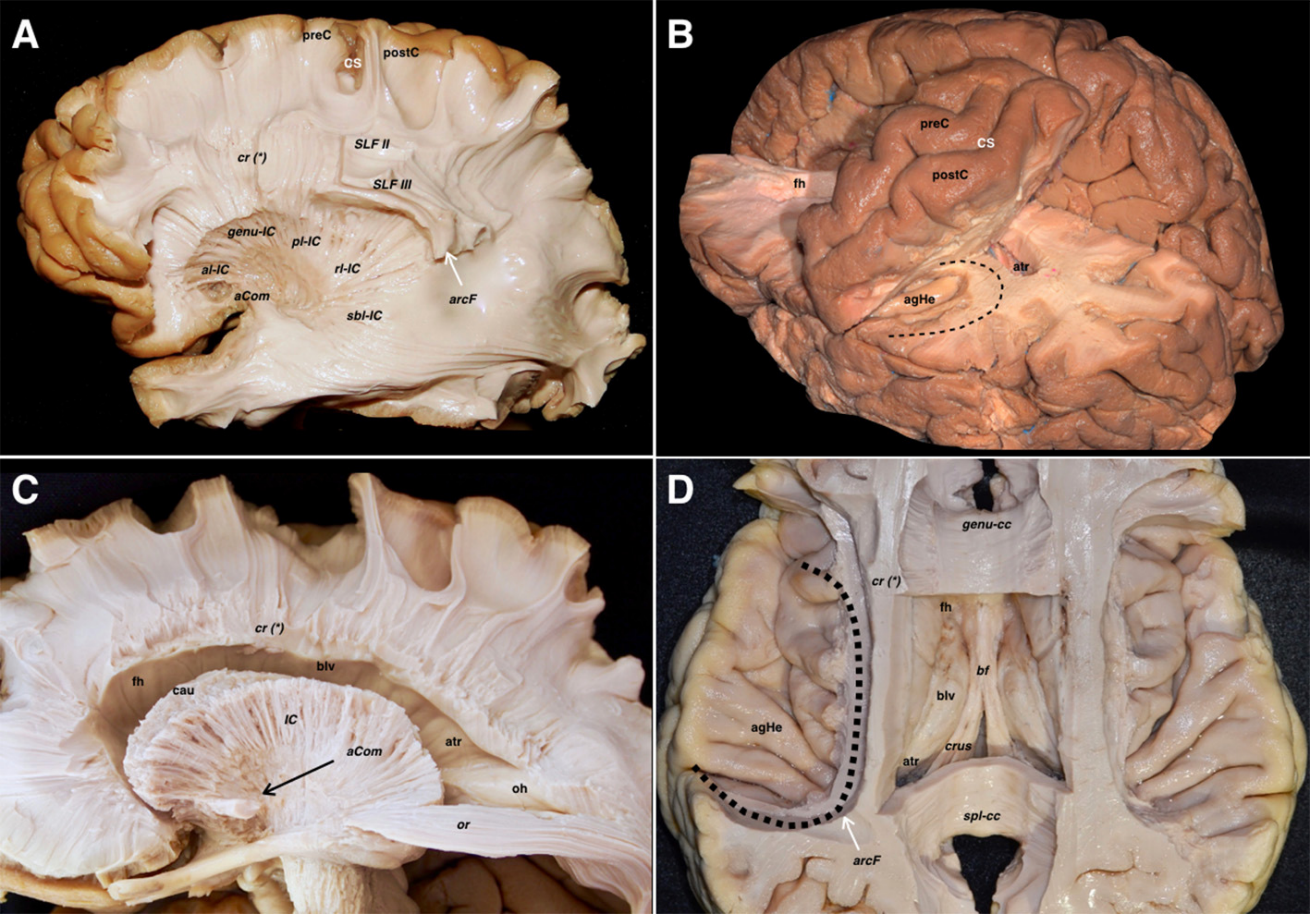
**(A)** The corona radiata is exposed in relation to the parietal parts of the SLF II, SLF III, and AF. The lateral basal ganglia are removed to show the different components of the IC namely the anterior limb, genu, posterior limb, retro-lenticular and sub-lenticular parts. The asterisk is showing the line where the projection (internal and external capsules) and commissural (corpus callosum) fibers of the corona radiata are intermingling. **(B)** Relationship of the central lobe with the lateral ventricle frontal horn and body. The central lobe fibers are mainly projection descending corticofugal and ascending thalamocortical. The different association tracts traversing the central lobe thickness are horizontally oriented and represented by the SLF I, SLF II, SLF III as well as the AF. The transverse gyrus of Heschl is located hidden in the depth of the sylvian fissure due to the telencephalic flexure and lobar movements. Note how the curve of the AF *(black dotted line)* wraps around the posterior insular point. **(C)** The IC and the corona radiata have been disconnected opening the lateral ventricle. The only projection fibers that have been preserved are the optic radiations. The AC is located ventral to the anterior limb of the IC. Note that the lateral aspect of the frontal horn, body, and atrium of the lateral ventricle has been uncovered to demonstrate the association, commissural, and projection tracts. The shape of most of the association fibers follows somehow the direction of the ventricular system, all influenced by the telencephalic flexure forces and movements. **(D)** An axial cut at the level of the telencephalic flexure has been performed, showing the hidden anatomy of the superior aspect of the temporal lobe. Interestingly, the representation of the AF shape and direction *(black dotted line)*, represents the forces that affect the human brain during its embryological development. In this superior view, a clear boundary in between the ventricular chambers and the lateral aspect of the brain is shown and represented by the corona radiata, at the point where the projection and commissural fibers join together. A wide window along the body of the corpus callosum has been created to show the body of the lateral ventricle from above. In its medial aspect, are shown most of the medial association fibers (mainly related to the limbic system) that assume a curved shape that is determined primarily by the telencephalic flexures. Most of them are connecting temporo-mesial with anterior frontal and septal areas. This fact explains the curved shape of the association fibers of the medial aspect that are found wrapping around the corpus callosum and the diencephalon. aCom, anterior commissure; agHe, anterior transverse gyrus of Heschl; al-ic, anterior limb of the internal capsule; arcF, arcuate fascicle; atr, atrium; bf, body of the fornix; blv, body of the lateral ventricle; cau, caudate nucleus; cr (*), corona radiata and callosal fibers intermingling point; crus, crus fornicis; CS, central sulcus; fh, frontal horn; genu-cc, corpus callosum genu; genu-IC, genu of the internal capsule; IC, internal capsule; oh, occipital horn; or, optic radiations; pl-IC, posterior limb of the internal capsule; postC, postcentral gyrus; preC, precentral gyrus; rl-IC, retrolenticular component of the internal capsule; sbl-IC, sublenticular component of the internal capsule; SLF II, superior longitudinal fasciculus II; SLF III, superior longitudinal fasciculus III; spl-cc, splenium of the corpus callosum.

The vertical SLF appeared connecting the supramarginal and angular with the superior and middle temporal gyri. Together with the tapetum, it represents one of the vertically oriented tracts in this area. SLF II-III and arcuate fascicle (AF) were then uncovered. SLF II-III was shown oriented in an anteroposterior linear-direction-traversing the central lobe. The AF was “non-longitudinally oriented” into this area and wrapped around the posterior insular point, connecting the posterior aspect of superior and middle temporal gyri with the central and frontal lobes. This tract is anteroposterior on its frontal aspect, but curves ventrally and laterally on its parietal aspect, thus lining up with the craniocaudal and lateromedial planes. The middle longitudinal fascicle (MLF) was found running anteroposteriorly from the superior temporal gyrus (STG) deep to the AF, curving upwards on the craniocaudal plane toward the superior parietal lobe. The IFOF, connecting the frontal and occipital lobes, followed an anteroposterior direction. However, it moves laterally and basally from the frontal to the temporal aspect. Therefore, it was considered as a curved association tract ventral to the insula. It runs dorsal to the uncinate fasciculus (UF), which courses lateral to the anterior perforated substance. Both converge at the limen insulae, being difficult to be individualized in this area. Removing the putamen allows the identification of the anterior commissure (AC), in which the main axis follows the lateromedial plane by crossing the midline. However, its anterior and posterior-lateral extensions curve into the anteroposterior and craniocaudal planes to reach their respective destinations into the amygdala and temporooccipital cortex. On its posterior projection, the IFOF and AC cover the optic radiations (OR), which remain lateral to the lateral ventricle. Each OR passes below the lentiform as the core of the sublenticular IC and is integrated into the sagittal stratum together with the IFOF and AC. The OR represents a projection system connecting the thalamus and the primary visual area in the ipsilateral occipital lobe following the anteroposterior and lateromedial planes of the space. After removing the sagittal stratum, the tapetum (tap) was shown covering the atrial lateral ependyma running downward and forward. It connects the basal and lateral aspects of both occipital and temporal lobes. As the AC, it is a curved commissural tract aligned into the lateromedial, anteroposterior, and craniocaudal planes. The inferior longitudinal fasciculus (ILF) rests basally connecting the temporal pole to the dorsolateral occipital cortex in the anteroposterior plane (Figures 3, 4).

### Medial-to-Lateral Dissection

**Figure 5:**
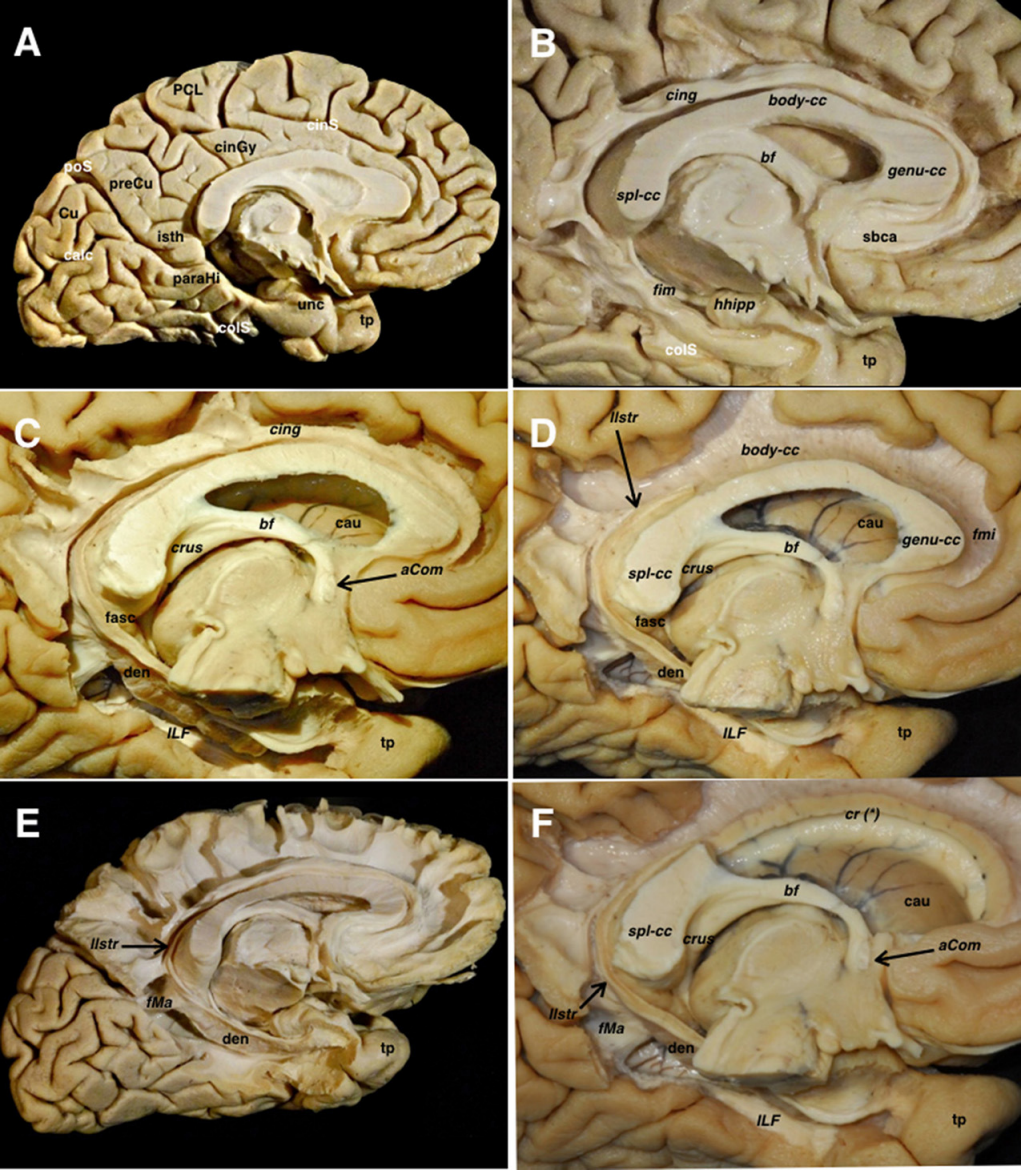
**(A)** A left hemisphere is viewed from its medial aspect. The telencephalic flexure is responsible for this complex shape of the human brain, where the medial and basal regions of the telencephalic vesicles (hemispheres) are finally covering the diencephalon and upper parts of the brainstem. The basal aspect might be considered part of the medial hemisphere due to its anatomical continuation. Indeed, the temporobasal, temporomesial regions, and temporal pole have reached their final locations due to the influences of the embryonic movements, thus encircling the thalamus and midbrain. This movement is also responsible for the complex orientation of the supratentorial ventricular system, and subsequently the arrangement of the white matter tracts (see also Figure 1). From a purely anatomical point of view, important midline surface landmarks are the cingulate sulcus with its marginal ascending ramus, the paracentral lobule and precuneus, the parieto- occipital fissure, the calcarine fissure, the splenium of CC, the parahippocampal gyrus, and the isthmus of the cingulum which is an anatomical key point at the junction of the calcarine and parietooccipital fissures. **(B)** The cortical layer covering the external limbic belt (cingulate and parahippocampal gyrus) has been removed exposing the whole cingulum and parahippocampal bundle. This curved association fiber tract mainly connects the subcallosal area and the mesial temporal pole, with some fibers to the precuneal region arising from the isthmus. Again, the telencephalic flexure is responsible for this curvilinear trajectory as the temporal pole is finally located ventral and anterior. **(C)** The parahippocampal fibers have been completely removed opening the atrium and showing the basal aspect of the dentate gyrus. The inferior longitudinal fascicle connecting the temporal and occipital lobes is shown in the floor of the temporal horn deep to the relative location of the fusiform gyrus. **(D)** The cingulum has been removed showing the callosal fibers from a medial view. The dentate gyrus moves posteriorly and superiorly and continues with the lateral longitudinal striae wrapping around the splenium of the corpus callosum. Note how this thin fiber bundle continues extraventricularly as the fasciola cinerea. This is considered as a rudiment of the hippocampal formation, and follows a curved trajectory around the corpus callosum to connect the fasciola and dentate gyrus with the induseum griseum on the dorsal surface of the callosal body. On the ventral surface of the hippocampus, the alveus continues posteriorly through the crus fornicis. Both crura become close at the midline at the ventral surface of the callosal splenium to run anteriorly through the body of the fornix along the floor of the lateral ventricle body. **(E)** I Once the body of the fornix reaches the foramen of Monro, it changes its direction to go inside the hypothalamic nuclei craneo-caudally to reach its target onto the mammillary body. A few fibers run anterior to the anterior commissure to reach the septal region, and are known as the precommissural part of the fornix. This is considered one of the intraventricular limbic belts, and its characteristic curved shape, is again a consequence of the flexure in the midline region. **(F)** The midline aspect of the rostrum, genu and body of the corpus callosum have been cut through the angle formed by the lateral wall and roof of the lateral ventricle. This is the point where the corona radiata and callosal fibers were seen intermingling in the lateral aspect of the hemisphere (Figure 4A). The medial hemispheric region shows a completely different organization compared to the lateral aspect of the hemisphere. The number of fiber systems are much less, and the organization is mainly provided by a wide association fascicle (cingulate- parahippocampal), which turns around the CC and the intraventricular temporomesial structures, and the underlying commissural fibers of the CC projecting toward the medial edge of the hemisphere. aCom, anterior commissure; bf, body of the fornix; body-cc, body of the corpus callosum; calc, calcarine fissure; cau, caudate nucleus; cing, cingulum; cinGy, cingulate gyrus; cinS, cingulate sulcus; colS, collateral sulcus; cr (*), corona radiata and callosal fibers intermingling point; Cu, cuneus; crus, crus fornicis; den, dentate gyrus; fasc, fasciola cinerea; fim, fimbria; fMa, forceps major; fmi, forceps minor; genu-cc, genu of the corpus callosum; hhipp, head of the hippocampus; ILF, inferior longitudinal fasciculus; isth, isthmus of the cingulum; llstr, lateral longitudinal stria; paraHi, parahippocampal gyrus; PCL, paracentral lobe; poS, parietooccipital sulcus; preCu, precuneus; sbca, subcallosal area; spl-cc, splenium of corpus callosum; tp, temporal pole; unc, uncus.

The cingulum, visualized after removing the cortex and u-fibers on the cingulate gyrus, emerges from the subcallosal area and runs posteriorly to join the isthmus. Ventral to the precuneus, some fibers emerge from the cingulum toward the medial aspect of the parietal lobe. The parahippocampal fibers represent the continuation of the cingulum toward the temporomesial region. The junction of the cingulum and parahippocampal fibers represents an extraventricular “C” shaped fiber system, belonging to the limbic system. Removing the cingulum and parahippocampal fibers, exposed the medial extension of the CC and the ventral aspect of the dentate gyrus. The lateral longitudinal stria was also seen dorsal to the CC and representing the dorsal remnant of the archicortex of the hippocampus, and fornix, showing connections to the amygdala and induseum griseum. All these limbic systems are represented in the anteroposterior, craniocaudal, and lateromedial planes (Figure 5).

### Transventricular Dissection

#### Lateral Aspect

**Figure 6:**
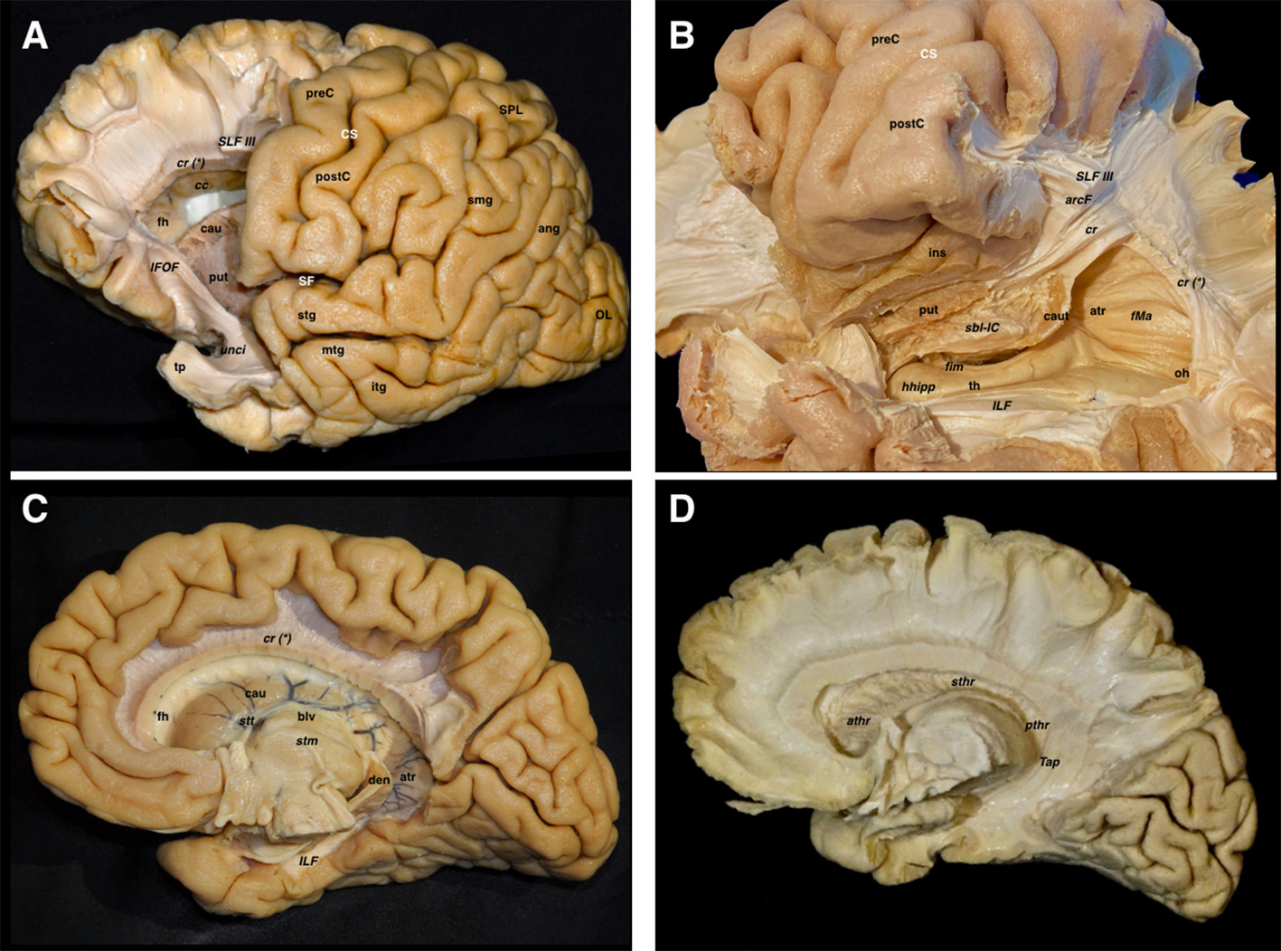
Both lateral and medial dissections end up reaching the last remaining fibers adjoining the walls of the lateral ventricle. **(A)** The frontal horn of the lateral ventricle is seen after opening its lateral wall. Thus, the last layer of fibers cut is the corticostriate and frontopontine descending fibers running to the anterior limb of the internal capsule, as well as the anterior thalamic radiations running from the anterior thalamic nuclei toward the prefrontal and cingular cortex. The SLF II & SLF III have been also cut in a more lateral plane. In the frontal region, the association fibers are purely longitudinal and oriented in the anteroposterior plane, while the projection fibers are also longitudinal but following a craneo-caudal direction. The curvilinear irregular shape of the IFOF and the uncinate are clearly seen in the ventral aspect of the telencephalic flexure. **(B)** In this image, the dissection has progressed into the temporoparietal area, which represents the dorsal and posterior parts of the telencephalic flexure. The vertical component of SLF, arcuate, corona radiate, sagittal stratum, and tapetum fiber bundles, as well as the ependyma of the atrium and temporal horn, have been removed, thereby providing a lateral view of the temporal horn and atrium. The head of the hippocampus is seen in the most anterior aspect of the temporal horn. The fimbria above the hippocampal surface follows a posterior direction in close contact with the choroidal fissure. The tail of the caudate nucleus is located in the temporal horn roof as well as in the anterior wall of the atrium while wrapping around the thalamus. This curved shape to the caudate follows the main dogma of the already discussed flexure. Note how the sub-lenticular component of the internal capsule is located ventral and medial to the putamen, where it runs lateral to the caudate. **(C)** A medial view of the lateral ventricle is shown in this dissection, where the cingulum, parahippocampal and callosal fibers, as well as the fornix have been removed. The curved shape of the caudate nucleus, as well as the stria terminalis connecting the amygdala and the septal area, are shown wrapping around the thalamus. **(D)** The lateral ependymal layer and caudate nucleus gray matter have been removed with the aim of uncovering the medial aspect of the internal capsule, with the anterior, superior, and posterior thalamic radiations, and the tapetum covering the lateral wall of the atrium, occipital and temporal horns. ang, angular gyrus; arcF, arcuate fascicle; athr, anterior thalamic radiation; atr, atrium; blv, body of the lateral ventricle; cau, caudate nucleus; caut, caudate nucleus tail; cc, corpus callosum; cr, corona radiata; cr (*), corona radiata and callosal fibers intermingling point; CS, central sulcus; den, dentate gyrus; fh, frontal horn; fim, fimbria; fMa, forceps major; hhipp, head of the hippocampus; IFOF, inferior frontooccipital fasciculus; ILF, inferior longitudinal fasciculus; ins, insula; itg, inferior temporal gyrus; mtg, middle temporal gyrus; oh, occipital horn; OL, occipital lobe; postC, postcentral gyrus; preC, precentral gyrus; pthr, posterior thalamic radiation; put, putamen; sbl-IC, sublenticular component of the internal capsule; SF, Sylvian fissure; SLF III, superior longitudinal fasciculus III; smg, supramarginal gyrus; SPL, superior parietal lobe; stg, superior temporal gyrus; sthr, superior thalamic radiation; stm, stria medullaris; stt, stria terminalis; Tap, tapetum; th, temporal horn; tp, temporal pole; unci, uncinate fascicle.

The IC and the corona radiata are in close relationship with the ventricular system. The anterior limb is related to the frontal horn, the genu to the foramen of Monro, the posterior limb to the body of the lateral ventricle, while the retro- and sublenticular components are related to the atrium and temporal/occipital horns. The floor of the frontal horn is formed by the rostrum of the CC, and the roof of the frontal horn and body is formed by the body CC. The splenial projections cover the medial wall and roof of the atrium. The head of the hippocampus is seen facing the uncal recess anteriorly and the amygdala superiorly. It runs anteroposteriorly, and the alveus becomes the fimbria of the fornix, which wraps around the pulvinar thalami in a superomedial direction, being thus represented in all three planes (Figure 6).

#### Medial Aspect

The caudate nucleus is seen in the ventricular wall. Scraping it away, allowed us to see the thalamic projections of the IC. The tapetum of the CC was seen covering the roof and lateral wall of the atrium from its medial aspect. The alveus and fimbria continue with the crus fornicis, which runs toward the midline, and then move forward, named body of the fornix, to surround the foramen of Monro and descend toward the mammillary bodies into the hypothalamus through the columns of the fornix.

### Cases Illustrations

#### Intra-axial Tumor Surgery in the Region of the Telencephalic Flexure

**Figure 7:**
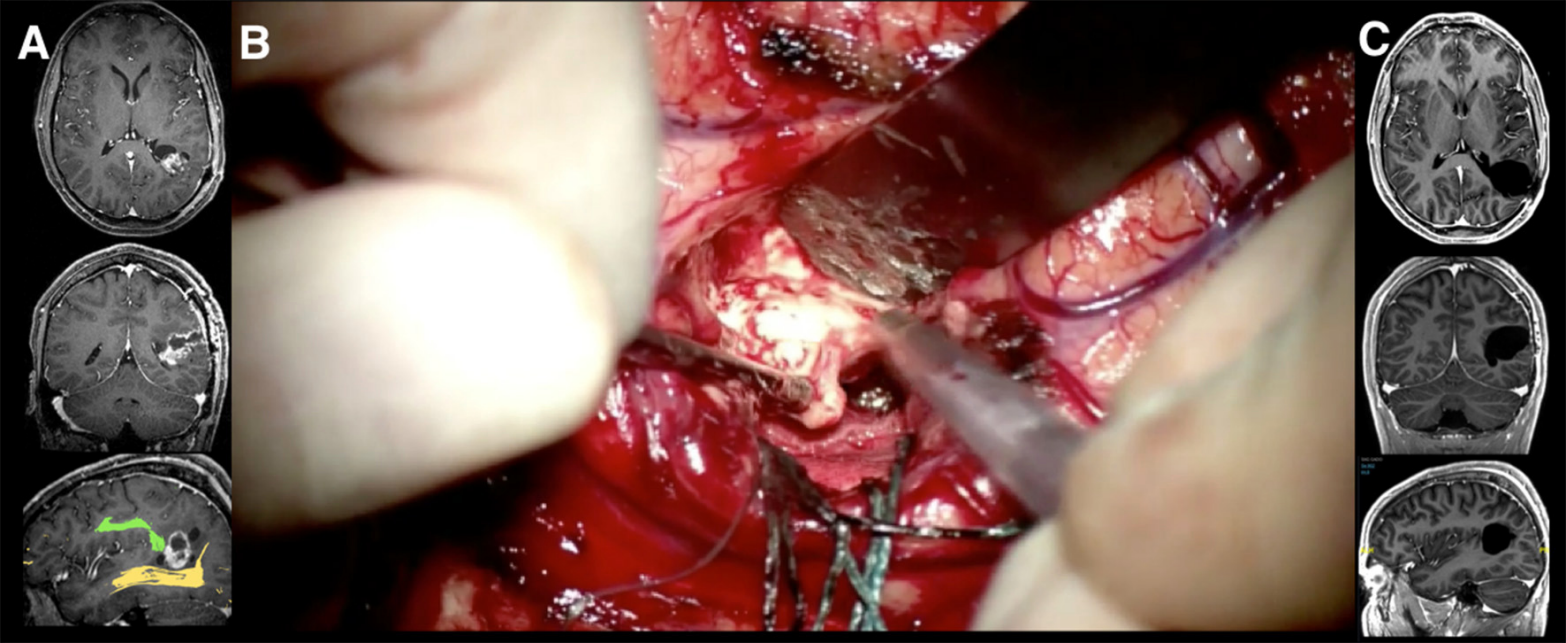
A 33-year-old man who presented with a headache was treated by awake surgery for a Pleomorphic Xanthoastrocytoma after an open biopsy was performed in a referral center. **(A)** The T1 weighted MR images after gadolinium injection in axial, coronal, and sagittal planes are shown. The lesion is located in the left temporoparietal area, just dorsal to the telencephalic flexure. This area shows highly eloquent cortical regions around the terminal part of the Sylvian fissure. The tumor grows from the cortical surface of the angular gyrus toward the ependymal wall of the left atrium. This area presents an underlying complex network of white matter tracts, the intimate relationships of which must be studied preoperatively. Some of these fibers show a curved shape as a result of the telencephalic flexure appearing due to the physical and genetic forces that made our brains more functionally advanced. Thus, the sagittal image shows its close relationship with the fused DTI-tractography images of the arcuate fascicle *(green)* and the optic radiations *(yellow*), which seemed to be displaced by the tumor itself. The neurological examination was completely normal including the visual field campimetry **(B)**. Due to the age of the patient and tumor location, an awake craniotomy was performed with the aim of testing the language and visual function during the tumor resection. The speech was tested to increase the tumor resection anteriorly and no deficit was noted even when reaching the most anterior part. During resection of the deeper parts of the tumor, intra- operative visual tests were performed. Stimulations to different points along the optic radiations evoked reversible scotomas in different portions of the visual field as described by the patient. At the completion of the optic radiation mapping, the resection of the tumor was performed radically in those areas where the vision remained normal despite subcortical stimulation. **(C)** The postoperative MRI at three months revealed a complete resection of the tumor. Postoperative campimetry at three months revealed a minor homonymous deficit in the right inferior quadrant.

One of the more challenging human brain areas to be surgically approached is the dominant fronto-temporoparietal junction. This area shows highly eloquent cortical regions around the terminal part of the Sylvian fissure. Moreover, the underlying white matter fiber tracts connecting these areas and also some other deep fascicles running in between them, make it especially important to understand the 3D architecture of this complex net of tracts. Dealing with an intra-axial tumor as a glioma in this fronto-temporoparietal region requires a strong understanding of its real subcortical origin and the surrounding eloquent areas, as well as the deep relationship of the relevant fiber tracts to the glioma itself. If we analyze all the fibers that are situated in between the lateral neocortex of the fronto-temporoparietal region and the ependyma of the body, atrium, and temporal horn of the lateral ventricle, several relevant fibers will appear (vertical SLF, arcuate fascicle, MLF, IFOF, anterior commissure, optic radiations, and tapetum). The sagittal stratum deserves a special mention, as a complex anatomical fibers arrangement into this area, mainly composed by the MLF, IFOF, OR, and other posterior thalamic radiations directed to non-visual areas of the cerebral cortex. In addition, small contributions to the sagittal stratum come from the anterior commissure anteriorly and the inferior longitudinal fasciculus inferiorly (4). As previously discussed, some of these fibers show a curved shape as a result of the telencephalic flexure appearing due to the physical and genetic forces that made our brains more functionally advanced. Advances in the field of neuroimaging (DTI, fMRI, neuronavigation, etc.) are helpful technologies in order to understand these fibers and their relationships. However, intraoperative testing and checking the functions of most of these structures is still the gold-standard technique to maximize the tumor resection minimizing postoperative deficits (Figure 7).

#### Disconnective Epilepsy Surgery of the Region

**Figure 8:**
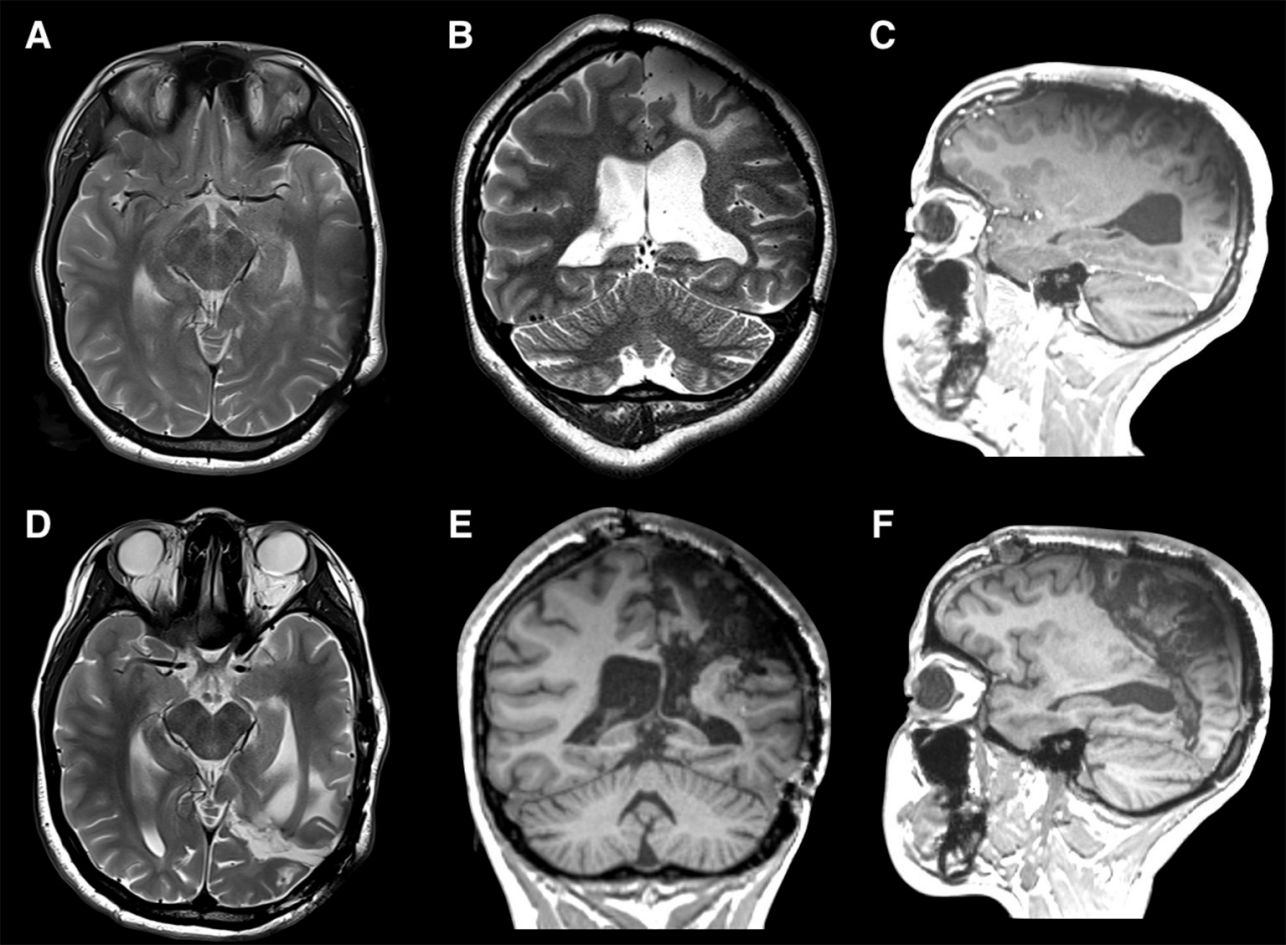
An 18-year-old left-handed girl presented with medically refractory epilepsy that was symptomatic since the age of 3 years due to a perinatal stroke in the territory of the left middle cerebral artery. Cerebral MRI (multiplanar) confirmed the radiological sequelae of an ischemic lesion involving primarily the central, parietal, and occipital lobes **(A–C)**. At the age of 7 years, based on phase II subdural grid evaluation, the majority of the seizures were determined to arise from the supplementary motor area (SMA) on the left side for which she underwent a subpial resection of the SMA. She continued to have an unfavorable seizure outcome and regression of developmental milestones along with a mild right hemiparesis with a limited fine motor movement of the right hand. Phase I and II (SEEG) evaluations showed major epileptogenic foci in the left parasagittal parietal lobe and precentral area. When the centro-parieto-occipital lobes (central quadrant) are involved, in the presence of normal frontal and temporal lobes, the situation becomes complex for a curative surgical procedure. A precise knowledge of the tracts that could traverse the lines of disconnection is mandatory. The association fibers like the SLF I, II, and III, IFOF, MLF, and cingulum are to be sectioned in addition to disconnecting the occipital lobe from the functional temporal lobe. The corticospinal, thalamocortical, parieto-pontine, occipito-pontine and optic radiations represent the projection fibers disconnected. The posterior lateral extension of the anterior commissure, tapetum, posterior third of corpus callosum, and splenium are the commissural fibers that are disconnected in this surgery. In view of the refractory nature of the epileptic illness and progressive cognitive decline, we decided to perform a centro-parieto-occipital lobotomy (central quadrantotomy). During this lobotomy, the arcuate fasciculus was spared, by performing a posteriorly directed oblique white matter section trajectory at the posterior insular point. Indeed, the direction of this disconnective procedure follows the direction of the forces that cause the telencephalic flexure during the early embryological development. The language functions remained unchanged and similar to the pre-operatively acquired language skills. The patient remained in Engel class I at the last follow-up at 10 months after surgery. Postoperative MRI (multiplanar) demonstrated the complete disconnections (arrows) of the central quadrant with preservation of the AF **(D–F)**.

Intractable epilepsy arising from the centro-parieto-occipital lobes is rare and is frequently a disease of childhood. The common etiologies are perinatal ischemic lesions, Sturge Weber syndrome, Rasmussen's encephalitis, and sub-hemispheric cortical dysplasias. When these etiologies involve the entire hemisphere, the surgery indicated is hemispherotomy (5–7). In the presence of normal frontal and temporal lobes, the situation becomes complex for a curative surgical procedure. In situations where the motor function is normal and near normal, investigations to locate the functional primary motor area needs to be done to ensure that the procedure planned for does not produce an irreversible motor deficit. In the dominant hemisphere, the locations of the speech areas need to be ascertained using fMRI. If then a disconnection of these lobes is deemed possible, a precise knowledge of the tracts that could traverse the lines of disconnection is mandatory. The association fibers as the SLF I, II, and III, IFOF, MLF, cingulum are to be sectioned in addition to disconnecting the occipital lobe from the functional temporal lobe. The corticospinal, thalamocortical, parietopontine, occipitopontine, and optic radiations represent the projection fibers disconnected. The lateral extension of the anterior commissure, tapetum, posterior third of corpus callosum, and splenium are the commissural fibers that are disconnected in this surgery. The arcuate fasciculus can be spared if a posteriorly directed oblique white matter section trajectory can be performed around after the corticectomy at the posterior insular point. An accurate understanding of the 3D anatomical configuration of the telencephalic flexure is primordial to know the distribution of these white matter tracts in order to achieve a total disconnection of the lobes (to achieve seizure freedom) while preserving important adjacent tracts in the frontal and temporal lobes and those tracts that traverse the telencephalic flexure (Figure 8).

## Discussion

The development of the cerebral cortex takes place predominantly in the fetal period. At approximately 25 days, the cephalic flexure divides the unfused neural folds into prosencephalon, mesencephalon, and rhombencephalon (3). At 4.5 gestational weeks (gw), the prosencephalic vesicle thins in the sagittal midline, growing in a mediolateral direction, resulting in forebrain outpouchings that will form the two telencephalic hemispheres. At 8 gw, the telencephalic hemispheres grow caudally bending in a ventral and rostral direction, best described as “C-shaped” (3). Perhaps counterintuitively, the posterior pole of the primitive telencephalon becomes the temporal lobe, while the occipital lobe is derived from the dorsal wall of this primitive telencephalon. This folding of the telencephalic hemispheres (telencephalic flexure) becomes the operculum and the future Sylvian fissure (8). The frontal and temporal lips of the Sylvian fissure, along with the insula all derive from the ventral margin of the primitive telencephalon (8). The first visualization of a fissure at this level starts at the 13 gw, perpendicular to the ventral surface of the hemisphere. This groove will become the posteroinferior peri-insular sulcus. At 18–19 gw the peri- insular sulcus is completed due to the faster development of the surrounding lobules compared with the growth speed of the insula. Around 20 gw, the opercularization process starts, which will develop into operculi completely covering the insula (9). The opercularization starts in the posterior part of the fissure as the parietotemporal cortex develops earlier than the frontal. The sylvian fissure completely closes at the end of the first postnatal year. The positional change of the hippocampus starts before the telencephalic flexure begins to form and its position is in part induced by the flexure, with the original dorsal aspect becoming its ventral aspect, and the dorsal area moving to a caudal end as a ventral structure after the “rotation”. These rotational movements of the hippocampal rudiment occur due to gradients induced by genetic expression, the fact that is key in some malformations (8). The formation of the forebrain fissures is also impacted by the development of the ventricles, which behaves as an external mechanical force rather than an internal force (8, 10, 11).

**Figure 9:**
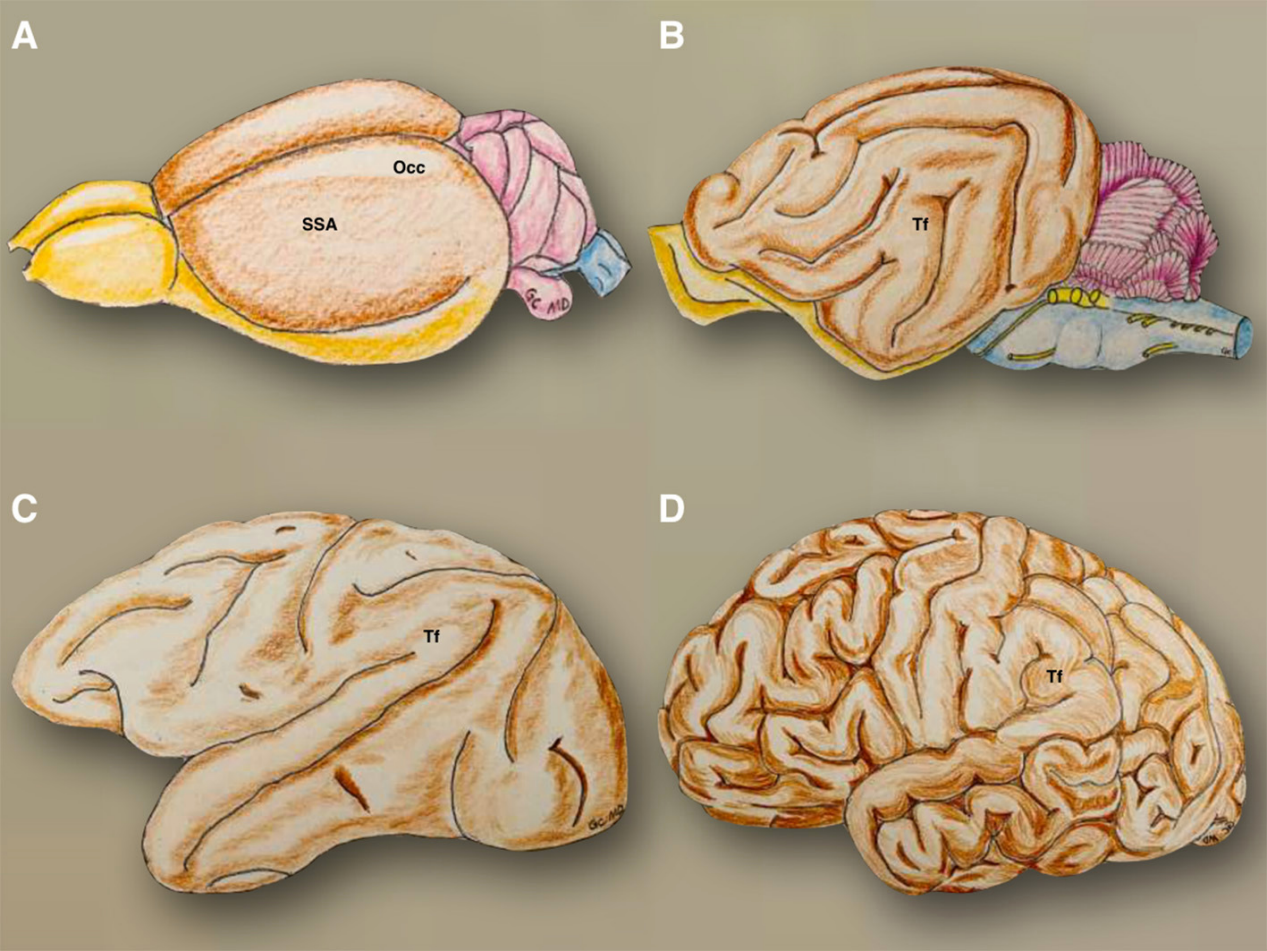
The phylogenetic evolution of the telencephalic flexure is illustrated. In the mouse **(A)**, the olfactory bulbs *(yellow)* are well developed and were continued posteriorly through the olfactory tracts and tubercles, to reach the entorhinal area on the posteroventral side of the hemispheres. The hemispheres are otherwise smooth *(brown)*, with the precentral area situated anteriorly to the somatosensory area, and postcentral region situated dorsally, and the occipital area located on the posterodorsal side. The cerebellum and brainstem are colored in *violet* and *blue*, respectively. In the cat **(B)**, the olfactory bulbs and tracts are situated on the ventral side of the hemispheres and connect posteriorly with the piriform lobe *(yellow)*. The telencephalic flexure is present in a primordial form, along with gyri and sulci *(brown)*. The cerebellum and brainstem are colored in *violet* and *blue*, respectively. In the Rhesus monkey **(C)**, the morphology of the telencephalic flexure is more similar but less pronounced to that of the human brain **(D)**. SSA, somatosensory and postcentral region; Occ, occipital area; Tf, telencephalic flexure.

The specific developmental patterns happening during its embryological evolution can be extrapolated to the phylogenetic changes of the CNS of different species (12, 13). From a morphological perspective, the evolution of the CNS in different species has gone from very simple tubular longitudinal shapes to more complex, round, and flexed forms in primates (Figure 1). A cross-species comparison of neuroembryology (Figure 9), with a particular focus on telencephalic flexure, reveals interesting information. In rodents, there is no formation of a telencephalic flexure, but, as in humans, their primary visual cortex lies on the dorsomedial aspect, rostral to the occipital pole. Furthermore, there is no looping of the stria terminalis, and the hippocampus extends all the way to the posterior hemisphere (14). Animals that do form a telencephalic flexure include all primates, dogs, whales, cats, cows, horses, and pigs (8). The real cause of the telencephalic folding or cortex gyrification has been hypothesized to be due to the lack of space for cortical development or due to differential regional proliferation and mechanical tension along the axons that try to bring together distal structures (15). The hippocampal shift from dorsal to a ventral position, and a reversal in orientation of genetic gradients are partly mediated by the telencephalic flexure (8). Seemingly this occurs at a marginally earlier embryonic stage in humans as compared with non-human primates.

When analyzing the differential proliferation speeds, the diverse origins of the operculum and the insular cortex must be considered. The operculi originate due to the radial migration from the ventricular and subventricular zones while the insula is formed by cells from the pallial/subpallial boundary that migrate around the basal ganglia. At 11–12 gw the Sylvian fossa is identified, and its configuration starts developing thanks to the curvature of the lateral ventricles. Since 13 gw, cells from the subpallial neurons migrate into the insular cortex along the radial glial fascicle reaching the final configuration before birth (16). Mallela et al. (17, 18) found different gene expressions from the opercula to the insula resulting in the highest proliferation of the former allowing their growth over the insula. This different origin might explain the fact that the tracts that developed later connecting frontal, temporal, and parietooccipital regions ‘avoid crossing the insula', thus being located ventral or dorsal to it, and in some cases wrapping around it with specific curved patterns (19–21).

There is still a large gap in understanding the excitatory and inhibitory signals mediating the formation of such a complex net of fibers, which will finally lead to the capricious human subcortical anatomy. In this sense, it has been recently discovered the role of the extracellular proteoglycan molecule keratan sulfate, which surrounds and envelops white matter tracts and fascicles from mid-gestation (much sooner than myelination of axons) and thus preserves the functional and anatomical “purity” of such fascicles, so that axons entering the tracts at one end cannot exist until they reach their destination and that axons from subcortical gray matter or indeed even other regions of cortex cannot enter the en-route tracts (8).

### Understanding Evolution of White Matter Tracts

Clear differences are observed when the subcortical architecture in monkeys is compared to the human one. Regarding the frontal connections, most of the studies reveal that monkeys' IFOF lacks the posterior projections to the occipital lobe ending at the temporal lobe level (22). Regarding the human brain's embryological development, the IFOF is one of the latest fascicles to develop, not appearing clearly identifiable till 20 gw (11). This longitudinally oriented tract is located ventral to the telencephalic bending, so it looks like not being too much affected by the flexure. However, the large development of the ventral insula pushed it downwards and maybe thickened its main core, marking its curved shape in this area. Although showing a similar structure to humans, monkeys' SLF cortical connections are slightly different. This fact is especially important for the SLF III, as it has the strongest and more anterior connections in the human inferior frontal gyrus, while the monkey's main connections are centered in the ventral premotor cortex. This is translated into a more important prefrontal connection in humans, especially on the right side. (23). Moreover, and although the horizontal segment is widely similar in humans and monkeys, the vertical segment is missed in the latter. The frontotemporal connections are the first ones to be identified during the embryogenesis (around the 17 gw), while the parietal branches appear a few weeks later during the development (20 gw) (11). SLF II and III posterior extensions to the angular and supramarginal gyri show a slight inferolateral curve, probably given by the inferior parietal lobe ventrolateral movement during the CNS development. The vertical component of SLF joins the superior parietal lobe (dorsal) with the posterior aspect of the superior temporal gyrus, which, in less developed species and the early embryonic stages, are oriented into the same horizontal-longitudinal axes. The ventral movement of the temporal lobe might be responsible for this vertical direction (Table 1).

**Table 1:** Summary of white matter tracts encountered during the lateral-to-medial and medial-to-lateral dissection.

**White matter tract**		**Type**	**Shape**	**Orientation**	**Connections**
**Lateral** **aspect**	**SLF I**	Association	Longitudinal	AP	SFG-SPL
	**SLF II**	Association	Longitudinal	AP	MFG-AG
	**SLF III**	Association	Longitudinal	AP	IFG-SMG
	**vcSLF**	Association	Longitudinal	CC	post.1/3 STG-SPL
	**Arcuate**	Association	Curved	AP/CC/LM	IFG-STG
	**MLF**	Association	Curved	AP/CC	mid.1/3 STG-SPL
	**IFOF**	Association	Curved	AP/CC	FBL-OL
	**ILF**	Association	Longitudinal	AP	TP-OL
	**Uncinate**	Association	Curved	AP/CC	FBL-TML
	**Ant. Commissure**	Commissural	Curved	AP/CC/LM	TML-TOL bilat.
	**Optic radiations**	Projection	Curved	LM/AP	LGB-OL
	**IC/cr**	Projection	Longitudinal	CC	Cortex-(BG,Tha,Brst,SC)
	**Corpus callosum**	Commissural	Curved	AP/CC/LM	Both hemispheres
**Medial** **aspect**	**Cingulum**	Association	Curved	AP/CC/LM	Subcallosal area -Parahippocampal
	**Fornix**	Association	Curved	AP/CC/LM	Hippoc.-MB
	**Lateral long. stria**	Association	Curved	AP/CC/LM	FC-indusium griseum
	**Stria terminalis**	Association	Curved	AP/CC/LM	Amygdala-septal area
	**Stria medularis**	Association	Curved	AP/CC	Habenula-septal & hypothal. Nuclei

*Their shape (longitudinal/curved), orientation/main axis (antero-posterior, craneo-caudal, latero-medial) and principal connections are detailed. AG, angular gyrus; AP, antero-posterior; BG, basal ganglia; Brst, brainstem; CC, cranio-caudal; cr, corona radiata; FBL, fronto-basal lobe; FC, fasciola cinerea; IC, internal capsule; IFG, inferior frontal gyrus; IFOF, inferior fronto-occipital fascicle; LGB, lateral geniculate body; LM, latero-medial; MB, mammillary body; MFG, middle fontal gyrus; MLF, middle longitudinal fasciculus; OL, occipital lobe; SC, spinal cord; SFG, superior frontal gyrus; SLF I, superior longitudinal fasciculus I; SLF II, superior longitudinal fasciculus II; SLF III, superior longitudinal fasciculus III; SMG, supramarginal gyrus; SPL, superior parietal lobe; STG, superior temporal gyrus; TML, temporomesial lobe; TOL, temporo-occipital lobes; TP, temporal pole; Tha, thalamus; vcSLF, vertical component of SLF*.

The SLF I anteroposterior longitudinal shape seems not to be affected by the flexure itself as the implicated cortical areas remain dorsal during the telencephalic bending.

The MLF shows a posterior and dorsal curve as joins the superior temporal gyrus with the superior temporal lobe. Before the tremendous telencephalic development happens, the rudimental temporal lobe is situated posterior and lateral to the parietal lobe, so probably, during this early stage these connections would be running horizontally, but anterior and dorsal from the temporal to the parietal rudiments.

Regarding the ILF, and according to the connected cortical areas (temporal pole and occipital lobe), the rudimental axons of this tract should have probably turned 180° as the temporal appears initially dorsal to the occipital lobe.

The human AF shows an increased number of temporal lobe connections, being responsible for a wider language specialization. When compared with macaques, we observe a longitudinal tract without a large number of temporal connections, mainly connecting the frontal and parietal cortices (24, 25). The ventrolateral movement of the neocortex of the temporal lobe is responsible for the C shape of the arcuate fascicle, as it is connecting the inferior frontal and superior temporal gyri, which were previously aligned in the same ‘x’ horizontal axis. The embryological development of such a highly specialized fiber tract is quite special, as it is not clearly visible until the 30 gw, being fully evident around the second year of age. Some pathological conditions during the Sylvian fissure development such as lissencephaly, polymicrogyria (26), or Perisylvian Syndrome (27), are related to alterations of development or even the absence of the AF. Most of the aforementioned tracts are located in between interconnected structures that initially were adjacent, becoming more distant as the telencephalic flexure proceeds, requiring their pathways to elongate and curve into a loop (8).

The changes suffered by the uncinate are the same suffered by the arcuate. Indeed, both are connecting temporal and frontal regions, with a big difference, as the uncinate connects the most rudimental areas of the human cortex (frontoorbital and temporomesial), related to the olfactory and mnesic functions, while the arcuate is related to one of the most advanced and complex human functions as speech is. Thus, the bending forces and the intrinsic anatomy of these different archicortical and neocortical areas are progressively separated by the appearance and development of the insular lobe, which will finally be responsible to push the uncinate to a ventral and the arcuate to a dorsal position into the telencephalic flexure.

Most of the relevant fibers in the medial aspect (cingulum, fornix. lateral longitudinal stria, stria terminalis, stria medularis), show a similar “C-shape” wrapping around the corpus callosum, ventricular system, and thalamus. This shape is the result of the telencephalic flexure, as most of these fiber tracts are longitudinally oriented in the first stages of development (11). One illustrative disease is the alobar and semilobar forms of holoprosencephaly where telencephalic fissure is absent due to uncleavage of the interhemisferic fissure. This causes the absence of temporal lobe rotation, becoming the most posterior aspect of the hemispheres, and therefore, the hippocampus does not rotate, having a linear disposition in the medial aspect, as it happens in rodents. This fact could explain a direct relationship between the telencephalic flexure and the curved shape of some of these tracts (8).

The internal capsule, and its cortical ascending and descending projections, are probably the fibers most affected by the telencephalic growth in primates. While the genu, anterior and posterior limbs are somehow oriented in the same sagittal plane, the aforementioned ventrolateral movement of the temporal lobe influences the appearing of the sub- and retrolenticular fibers in a more lateral position, curving its direction posteriorly to the temporal and occipital lobes respectively, and covering the lateral wall of the temporal horn, atrium and occipital horn of the lateral ventricles. Thus, the final shape of the internal capsule is a reminder of a baseball glove hugging the lenticular core.

A relevant part of the internal capsule, regarding these specific changes in shape and direction, is the optic radiations. As thalamic corticopetal fibers, they run from the lateral geniculate toward the occipital lobe in a horizontal longitudinal direction. The visual projection system runs dorsally and superiorly in fishes and even in rodents to reach the dorsally located occipital region regarding the central position of the thalamus (28). However, and probably due to the expansive lateral growing of the lateral ventricles and the telencephalic bending in primates, the occipital lobe is moved to the posterior pole of the brain. Therefore, the optic radiations are pushed inferiorly and laterally being forced to travel through the depth of the temporal lobe on its way to the visual areas.

Important attention must be given to the commissural fibers. The anterior commissure is the primordial commissural tract, connecting both temporomesial (medial component) and temporo-occipital areas (lateral component). Its posteriorly directed shape might have happened due to the fragmentation of the primordium of the temporo-occipital cortex, the temporal areas moving ventral, anterior, and lateral, and the occipital ones moving dorsally. As the corpus callosum connects the neocortex of both telencephalic hemispheres, it perfectly describes the way in which the wide development of the neocortex has covered the central core. Thus, it shows a lateromedial direction in the coronal plane, as well as an anterior curve (genu-forceps minor) and a posterior one (splenium-forceps major) in the sagittal plane. These two curves contain fibers connecting basal and polar areas of the frontal lobe, as well as the medial and basal aspects of the occipital lobe respectively. A special part of the corpus callosum is the tapetum, which, as connects both temporal lobes, runs in a ventrolateral direction in the lateral wall of the atrium and temporal horn. The corpus callosum is mainly developed once the flexure has happened (8).

Performing the simple exercise of “drawing” these fibers in some animals' brains, one might realize that ‘older CNS’ (in phylogenetic terms), have more longitudinally oriented tracts (specifically in one plane). However, the primates' CNS evolution added the acquisition of more complex functions. This was the result of evolutional genetic expressions, giving rise to a brain in which the lobes are morphologically located in the three different planes due to the telencephalic movements (Figures 1, 9). These growth forces taking place in the human brain during its development, as well as the flexure that will hide the insular lobe, involve important implications for the 3D understanding of the white fibers (Figure 1).

Our dissections have shown the complex anatomical arrangement and relationships among the different association, projection, and commissural fiber tracts around the central core. Interestingly, we mainly found anteroposterior longitudinally oriented fibers in the frontal region. However, posteriorly (parietooccipital) and basally (temporal), some fibers appear curving around the flexure, ventrally (IFOF, UF, and AC), and dorsally (MLF, vertical SLF, and AF) (Figure 4). Some of those fiber tracts, especially those connecting relatively ‘distant’ cortical areas (AF, IFOF, UC) show a specific curved shape. Those tracts connecting adjacent lobes, predominantly show a longitudinal direction as the SLF, MLF, and ILF. This pattern of shapes could be related to the fact that the temporal lobe has been pushed laterally, anteriorly, and ventrally from its posterior position, and the occipital lobe has moved posteriorly from a dorsal location during the embryological development. On the other hand, the medial movement of the ventral cortex could be ascribed to the insula being pushed to the depth of the Sylvian fissure, being responsible for the curved shape of the aforementioned fascicles, as well as for the fan-shape radiations of the corona radiata (Table 1). Therefore, while there exists a clear relationship between the white matter tracts' disposition and the formation of the Sylvian fissure in humans, it is not yet known if this is the cause or the effect.

### Surgical Implications

Oncological surgery for intra-axial lesions deep to the perisylvian cortices, especially for the dominant hemisphere, requires a profound knowledge of the anatomy of the cortex and of the subjacent WMT, some of which are of curvilinear morphology and the others longitudinal. Analysis of pre-operative MR imaging (including DTI and fMRI) and its use for neuronavigation are standard techniques to allow maximal preservation of the WMT connectivity. The 3D anatomical appreciation of this region may be particularly complex and the performance of cadaveric dissection studies as well as case-to-case analysis of preoperative images to evaluate the distortion of the normal anatomy provoked by the tumor are essential to tailor the surgical procedure. To perform a maximally safe resection, intraoperative monitoring through the performance of awake craniotomies is often used to perform a direct study of the WMT and thereby obtain a functionally-guided resection.

The anatomy of this region is also of particular importance for the planning of sub- hemispheric epilepsy surgery (29–33). When dealing with lesions of the centro-parieto-occipital lobes (central quadrant), disconnective surgery implies particular problems because of the necessity to preserve some traversing fibers while selectively disconnecting all afferent and efferent connections of these lobes. White matter areas where the associative fibers are intermingling with projection and commissural fibers should be carefully analyzed to safely perform the disconnective epilepsy surgery in order to obtain a favorable seizure outcome with minimal additional neurological morbidity.

### Limitations of the Study and Future Perspectives

This cadaveric study defined the complex orientation of the white matter anatomy of this region in adult human brains. Ongoing development of diffusion tensor imaging techniques in human brains with the surface rendering of these important tracts around the flexure will help improve our understanding of the intimate relationships of the projection, association, and commissural fibers in this region. We have attempted to provide correlations from existing literature using human embryological data and cross-species studies of the region of the telencephalic flexure. However, this can be further enhanced with human embryological studies using histological and/or neuroimaging methods.

## Conclusions

A precise knowledge of the connectome of the region around the telencephalic flexure is crucial to safe surgery for intra-axial pathologies in the area. A 3D appreciation of the WMT topography allows the identification and preservation of eloquent tracts during the removal of intra-axial tumors in this region. This is also crucial while performing disconnective epilepsy surgery of the centro-parieto-occipital lobes that aim to achieve a complete disconnection while preserving important white matter tracts that adjoin or traverse the telencephalic flexure.

## Data Availability Statement

The original contributions presented in the study are included in the article/supplementary material, further inquiries can be directed to the corresponding author/s.

## Ethics Statement

Ethical review and approval was not required for the study on human participants in accordance with the local legislation and institutional requirements. The patients/participants provided their written informed consent to participate in this study.

## Author Contributions

PG-L and RD conceived the paper. PG-L and CG performed the cadaveric dissection. GC and PG-L analyzed the data and wrote the main body of the text. CT and JM wrote two paragraphs of the paper. EP performed an analysis and revision of the paper. MM and RD supervised the paper and both deserve to be the last authors. All authors revised the last version of the paper.

## Conflict of Interest

The authors declare that the research was conducted in the absence of any commercial or financial relationships that could be construed as a potential conflict ofinterest.

## Publisher's Note

All claims expressed in this article are solely those of the authors and do not necessarily represent those of their affiliated organizations, or those of the publisher, the editors and the reviewers. Any product that may be evaluated in this article, or claim that may be made by its manufacturer, is not guaranteed or endorsed by the publisher.

## References

[1] Ludwig E Klingler J: Der innere Bau des Gehirns dargestellt auf Grund makroskopischer Präparate in Atlas cerebri humani. Basel: Karger, (1956).

[2] Ture U Yasargil MG Friedman AH Al-Mefty Al-Mefty O: Fiber dissection technique: lateral aspect of the brain. Neurosurgery. (2000) 47:417–26. 10.1097/00006123-200008000-00028 10942015

[3] O'Rahilly R Muller F. Developmental stages in human embryos: revised and new measurements. Cells Tissues Organs. (2010) 192:73–84. 10.1159/000289817 20185898

[4] Maldonado IL Destrieux C Ribas EC Guimarães BS Cruz PP Duffau H. Composition and organization of the sagittal stratum in the human brain: a fiber dissection study. J Neurosurg. (2021) 135:1214–22. 10.3171/2020.7.JNS192846 33418529

[5] Daniel RT Joseph TP Gnanamuthu C Chandy MJ. Hemispherotomy for paediatric hemispheric epilepsy. Stereotact Funct Neurosurg. (2001) 77:219–22. 10.1159/000064609 12378079

[6] Daniel RT Villemure JG. Peri-insular hemispherotomy: potential pitfalls and avoidance of complications. Stereotact Funct Neurosurg. (2003) 80:22–7. 10.1159/000075155 14745204

[7] Villemure JG Daniel RT. Peri-insular hemispherotomy in paediatric epilepsy. Childs Nerv Syst. (2006) 22:967–81. 10.1007/s00381-006-0134-3 16804712

[8] Sarnat HB Flores-Sarnat L. Telencephalic Flexure and Malformations of the Lateral Cerebral (Sylvian) Fissure. Pediatr Neurol. (2016) 63:23–38. 10.1016/j.pediatrneurol.2016.05.005 27590993

[9] Afif A Bouvier R Buenerd A Trouillas J Mertens P. Development of the human fetal insular cortex: study of the gyration from 13 to 28 gestational weeks. Brain Struct Funct. (2007) 212:335–46. 10.1007/s00429-007-0161-1 17962979

[10] Gholipour A Rollins CK Velasco-Annis C Ouaalam A Akhondi-Asl A Afacan O. A normative spatiotemporal MRI atlas of the fetal brain for automatic segmentation and analysis of early brain growth. Sci Rep. (2017) 7:476. 10.1038/s41598-017-00525-w 28352082 PMC5428658

[11] Horgos B Mecea M Boer A Szabo B Buruiana A Stamatian F. White Matter Dissection of the Fetal Brain. Front Neuroanat. (2020) 14:584266. 10.3389/fnana.2020.584266 33071763 PMC7544931

[12] E. H: Ueber die Entwicklungstheorie Darwin's. In: Amtlicher Berich ueber die acht und dreissigste Versammlung Deutscher Naturforscher und aerzte. in Stettin.: Stettin: Hessenland's Buchdruckerei, (1864). pp. 17–30.

[13] Hall BK. Ontogeny does not recapitulate phylogeny, it creates phylogeny. In: Robert J. Richards ED e (ed): A review of The Tragic Sense of Life: Ernst Haeckel and the Struggle over Evolutionary Thought. (2011). Vol 13, pp 401–4. 10.1111/j.1525-142X.2011.00495.x

[14] Basma J Guley N Michael Ii LM Arnautovic K Boop F Sorenson J. The Evolutionary Development of the Brain As It Pertains to Neurosurgery. Cureus. (2020) 12:e6748. 10.7759/cureus.6748 32133270 PMC7034762

[15] Van Essen DC Donahue CJ Glasser MF. Development and evolution of cerebral and cerebellar cortex. Brain Behav Evol. (2018) 91:158–69. 10.1159/000489943 30099464 PMC6097530

[16] Gonzalez-Arnay E Gonzalez-Gomez M Meyer G. A Radial Glia Fascicle Leads Principal Neurons from the Pallial-Subpallial Boundary into the Developing Human Insula. Front Neuroanat. (2017) 11:111. 10.3389/fnana.2017.00111 29259547 PMC5723317

[17] Mallela AN Deng H Brisbin AK Bush A Goldschmidt E. Sylvian fissure development is linked to differential genetic expression in the pre-folded brain. Sci Rep. (2020) 10:14489. 10.1038/s41598-020-71535-4 32879369 PMC7468287

[18] Mallela AN Deng H Bush A Goldschmidt E. Different Principles Govern Different Scales of Brain Folding. Cereb Cortex. (2020) 30:4938–48. 10.1093/cercor/bhaa086 32347310

[19] Choi CY Han SR Yee GT Lee CH. A understanding of the temporal stem. J Korean Neurosurg Soc. (2010) 47:365–9. 10.3340/jkns.2010.47.5.365 20539796 PMC2883057

[20] Peltier J Verclytte S Delmaire C Pruvo JP Godefroy O Le Gars D. Microsurgical anatomy of the temporal stem: clinical relevance and correlations with diffusion tensor imaging fiber tracking. J Neurosurg. (2010) 112:1033–8. 10.3171/2009.6.JNS08132 19612976

[21] Ribas EC Yagmurlu K de Oliveira E Ribas GC Rhoton A. Microsurgical anatomy of the central core of the brain. J Neurosurg. (2018) 129:752–69. 10.3171/2017.5.JNS162897 29271710

[22] Thiebaut de. Schotten M, Dell'Acqua F, Valabregue R, Catani M: Monkey to human comparative anatomy of the frontal lobe association tracts. Cortex. (2012) 48:82–96. 10.1016/j.cortex.2011.10.001 22088488

[23] Hecht EE Gutman DA Bradley BA Preuss TM Stout D. Virtual dissection and comparative connectivity of the superior longitudinal fasciculus in chimpanzees and humans. Neuroimage. (2015) 108:124–37. 10.1016/j.neuroimage.2014.12.039 25534109 PMC4324003

[24] Ghazanfar AA. Language evolution: neural differences that make a difference. Nat Neurosci. (2008) 11:382–4. 10.1038/nn0408-382 18368042

[25] Rilling JK Glasser MF Preuss TM Ma X Zhao T Hu X. The evolution of the arcuate fasciculus revealed with comparative DTI. Nat Neurosci. (2008) 11:426–8. 10.1038/nn2072 18344993

[26] Andrade CS Figueiredo KG Valeriano C Mendoza M Valente KD Otaduy MC. DTI-based tractography of the arcuate fasciculus in patients with polymicrogyria and language disorders. Eur J Radiol. (2015) 84:2280–6. 10.1016/j.ejrad.2015.07.014 26216794

[27] Kilinc O Ekinci G Demirkol E Agan K. Bilateral agenesis of arcuate fasciculus demonstrated by fiber tractography in congenital bilateral perisylvian syndrome. Brain Dev. (2015) 37:352–5. 10.1016/j.braindev.2014.05.003 24852949

[28] Mueller T. What is the thalamus in zebrafish? Front Neurosci. (2012) 6:64. 10.3389/fnins.2012.00064 22586363 PMC3345571

[29] Cossu G Messerer M Lebon S Daniel RT. Posterior Peri-insular Quadrantotomy. In: Cataltepe O, Jallo GI. e (ed): Pediatric Epilepsy Surgery: Preoperative Assessment and Surgical Treatment. New York: Thieme, (2020). p. 465–71.

[30] Cossu G Gonzalez-Lopez P Pralong E Kalser J Messerer M Daniel RT. Unilateral prefrontal lobotomy for epilepsy: technique and surgical anatomy. Neurosurg Focus. (2020) 48:E10. 10.3171/2020.1.FOCUS19938 32234977

[31] Cossu G Messerer M Lebon S Pralong E Seeck M. R.T. D Anterior Peri-insular quadrantotomy. In Cataltepe O, Jallo GI. e (ed): Pediatric epilepsy Surgery: preoperative Assessment and Surgical Treatment. ed Second Edition New York: Thieme, (2020)., pp 459–464

[32] Daniel RT Meagher-Villemure K Farmer JP Andermann F Villemure JG. Posterior quadrantic epilepsy surgery: technical variants, surgical anatomy, and case series. Epilepsia. (2007) 48:1429–37. 10.1111/j.1528-1167.2007.01095.x 17441997

[33] Gonzalez-Lopez P Cossu G Pralong E Baldoncini M Messerer M Daniel RT. Anterior peri-insular quadrantotomy: a cadaveric white matter dissection study. J Neurosurg Pediatr. (2019) 25:331–9. 10.3171/2019.10.PEDS19472 31860823

